# Comparing supervised and unsupervised multiresolution segmentation approaches for extracting buildings from very high resolution imagery

**DOI:** 10.1016/j.isprsjprs.2014.07.002

**Published:** 2014-10

**Authors:** Mariana Belgiu, Lucian Drǎguţ

**Affiliations:** a Department of Geoinformatics – Z_GIS, Salzburg University, Schillerstr. 30, 5020 Salzburg, Austria; b West University of Timisoara, Department of Geography, Vasile Parvan Avenue, 300223 Timisoara, Romania

**Keywords:** Supervised segmentation, Unsupervised segmentation, OBIA, Buildings, Random forest classifier, OpenStreetMap

## Abstract

Although multiresolution segmentation (MRS) is a powerful technique for dealing with very high resolution imagery, some of the image objects that it generates do not match the geometries of the target objects, which reduces the classification accuracy. MRS can, however, be guided to produce results that approach the desired object geometry using either supervised or unsupervised approaches. Although some studies have suggested that a supervised approach is preferable, there has been no comparative evaluation of these two approaches. Therefore, in this study, we have compared supervised and unsupervised approaches to MRS. One supervised and two unsupervised segmentation methods were tested on three areas using QuickBird and WorldView-2 satellite imagery. The results were assessed using both segmentation evaluation methods and an accuracy assessment of the resulting building classifications. Thus, differences in the geometries of the image objects and in the potential to achieve satisfactory thematic accuracies were evaluated. The two approaches yielded remarkably similar classification results, with overall accuracies ranging from 82% to 86%. The performance of one of the unsupervised methods was unexpectedly similar to that of the supervised method; they identified almost identical scale parameters as being optimal for segmenting buildings, resulting in very similar geometries for the resulting image objects. The second unsupervised method produced very different image objects from the supervised method, but their classification accuracies were still very similar. The latter result was unexpected because, contrary to previously published findings, it suggests a high degree of independence between the segmentation results and classification accuracy. The results of this study have two important implications. The first is that object-based image analysis can be automated without sacrificing classification accuracy, and the second is that the previously accepted idea that classification is dependent on segmentation is challenged by our unexpected results, casting doubt on the value of pursuing ‘optimal segmentation’. Our results rather suggest that as long as under-segmentation remains at acceptable levels, imperfections in segmentation can be ruled out, so that a high level of classification accuracy can still be achieved.

## 1 Introduction

Over the last decade, object-based image analysis (OBIA) has become accepted as an efficient method for extracting detailed information from very high resolution (VHR) satellite imagery (Blaschke, 2010). The most critical step in OBIA is the segmentation of the imagery into spectrally homogeneous, contiguous image objects (Baatz and Schäpe, 2000, Benz et al., 2004). Segmentation algorithms ideally generate image objects that match the target objects, but in reality, segmentation remains an unresolved problem in OBIA (Arvor et al., 2013, Drǎguţ et al., 2010, Feitosa et al., 2006, Hay and Castilla, 2008, Hay et al., 2005). Segmentation results are greatly influenced by the image quality, the number of image bands, the image resolution and the complexity of the scene (Drăguţ et al., 2014, Fortin et al., 2000). Furthermore, most of the available segmentation algorithms need to be fine-tuned by the user to extract specific objects of interest (Hay et al., 2005), which means that image segmentation remains a highly subjective task usually achieved in a manual, trial-and-error fashion (Arvor et al., 2013, Hay et al., 2005, Meinel and Neubert, 2004).

A number of attempts have been made to develop methods for the objective identification of optimal segmentation parameters that are, at least to some degree, automatic (Anders et al., 2011, Drǎguţ et al., 2010, Esch et al., 2008, Feitosa et al., 2006, Maxwell and Zhang, 2005). Most of these methods have been designed for multiresolution segmentation (MRS), which is one of the most popular segmentation algorithms (Esch et al., 2008). By analogy with segmentation evaluation (Zhang et al., 2008, Zhang, 1996), these methods can be broadly classified as either supervised or unsupervised. Most of the methods involve performing multiple segmentations, which are then evaluated either to select the most suitable segmentation according to objective criteria or to select desirable image objects and further refine those that do not meet given criteria.

The supervised approaches require reference data with which to adjust the segmentation parameters so that the image objects best approximate the target objects. Image objects can be fitted to the reference data using a fuzzy logic approach (Maxwell and Zhang, 2005), by means of a genetic algorithm (Feitosa et al., 2006) or through a quantitative comparison of frequency distribution matrices (Anders et al., 2011).

The unsupervised approaches are purely data driven and use the image statistics to determine the optimal parameters for delineating image objects (e.g., using the estimation of scale parameter (ESP) method: (Drǎguţ et al., 2010)) or for optimizing image objects (e.g., using the segmentation optimization procedure (SOP): (Esch et al., 2008)).

The existing supervised and unsupervised segmentation methods have previously been applied and evaluated independently in various application scenarios. Several studies have assessed the performances of segmentation algorithms implemented in different software packages (Carleer et al., 2005, Marpu et al., 2010, Meinel and Neubert, 2004, Neubert and Herold, 2008, Neubert et al., 2006, Räsänen et al., 2013). However, to the best of our knowledge, there has been no study dedicated to a comparative evaluation of supervised and unsupervised segmentation methods, possibly because such methods have rarely ended up in operational tools. Supervised methods have generally been recommended for evaluating segmentation results if accurate ground truth data are available, as the resulting evaluation is believed to be more accurate (Hoover et al., 1996, Wanqing et al., 2004). Because classification accuracy was believed to be highly dependent on the quality of the segmentation, supervised segmentation methods were thought to lead to more accurate classifications (Gao et al., 2011, Ryherd and Woodcock, 1996). However, the differences between the results obtained from supervised and unsupervised methods have never been evaluated. Such an evaluation would be of considerable importance for any attempts to automate the OBIA segmentation process (Jakubowski et al., 2013), as it would reveal the degree to which classification accuracy is likely to be compromised by attempts to reduce the amount of user intervention required to set up the segmentation parameters.

The objective of this study was to compare supervised and unsupervised approaches for MRS. To achieve this objective, we made use of one supervised and two unsupervised segmentation methods that were either accessible in the public domain or available from their developers as operational tools. The two segmentation approaches were assessed using segmentation evaluation methods and by an accuracy assessment of the resulting building classifications. Thus, the differences in the geometries of the image objects and in their potential to produce satisfactory thematic accuracies were evaluated. This comparative study has focused on the delineation of bona fide objects (buildings) due to their unambiguous ontological status.

## 2 Study area and data

**Fig. 1:**
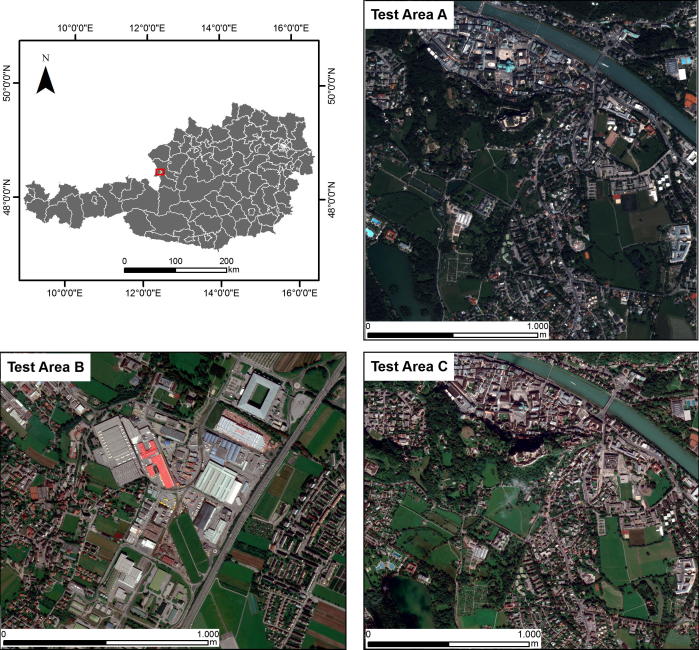
Location of the test areas in Salzburg, Austria. Images are displayed as a true color composition (RGB). (For interpretation of the references to colour in this figure legend, the reader is referred to the web version of this article.)

For this study, we used three test areas located in Salzburg, Austria (Table 1 and Fig. 1). Test Area A and Test Area C cover a dense residential area, whereas Test Area B covers an industrial area with dispersed residential houses. The available data used for the tests in these areas were pan-sharpened QuickBird and pan-sharpened WorldView-2 imagery. Test areas A and C cover the same part of the city but were investigated using different data sources to assess the sensitivity of the evaluated methods to different sensors.

**Table 1:** Summary of the three test areas and characteristics of the corresponding satellite imagery.

Test area	Imagery	Spatial resolution	Location	Dimensions (pixels)	Band composition
A	QuickBird	0.6	Salzburg city – down-town area	3300 × 3300	Blue, green, red, NIR
B	WorldView-2	0.5	Salzburg city – industrial area	3426 × 3211	Coastal blue, blue, green, yellow, red, red-edge, NIR1, NIR2
C	WorldView-2	0.5	Salzburg city – down-town area	4282 × 3875	As above

## 3 Methodology

In this study, the following methods have been evaluated and compared:

• the supervised segmentation method proposed by Anders et al. (2011) and
• the un-supervised segmentation methods proposed by Drăguţ et al. (2014) and Esch et al. (2008).

These three methods are all implemented as operational tools based on the MRS algorithm (Baatz and Schäpe, 2000). MRS is a bottom-up, region-merging technique that partitions the image into image objects on the basis of homogeneity criteria controlled by user-defined parameters such as shape, compactness/smoothness and scale parameter SP (Baatz and Schäpe, 2000).

While the MRS algorithm permits a multi-scale analysis of the target classes (Baatz and Schäpe, 2000), in this study, we evaluated only single-level segmentation procedures (i.e., relevant to a feature extraction approach) for the sake of compatibility in the comparisons because only the method developed by Drăguţ et al. (2014) generates multi-scale segmentation levels. The method proposed by Esch et al. (2008) performs multi-scale analysis but delivers only one segmentation level, whereas the supervised method proposed by Anders et al. (2011) selects a single scale out of multiple possibilities (without combining image objects across scales). Therefore, we adopted the common denominator, i.e., single-level segmentation. A brief description of the evaluated methods is provided in the following subsections.

### 3.1 Supervised and unsupervised segmentation methods

#### 3.1.1 Supervised segmentation approach

The only available supervised method was the segmentation accuracy assessment (SAA) method (Anders et al., 2011), which relies on reference samples to assess the segmentation accuracy of the image objects generated using different SPs. Frequency distribution matrices are calculated for the image objects at each segmentation level and compared with those of the corresponding reference objects. The appropriate segmentation level gives the lowest segmentation error (SE), which is calculated as the mean of the sum of absolute error (SAE) values (Anders et al., 2011). This method was implemented in the Python scripting environment.

Thirty reference polygons for two building classes (small buildings and large buildings) were randomly selected from OpenStreetMap (OSM) for training in the SAA method. The OSM buildings data were accessed using the download service offered by Geofabrik GmbH (http://download.geofabrik.de/). The geometries of the selected buildings were visually inspected and corrected where necessary. A series of image segmentation levels was generated on all image layers (image bands) using SPs of 10-500 at intervals of 10, and of 501 and 700. The image objects and reference samples were overlaid to evaluate the frequency distribution matrices and to calculate the segmentation error. The segmentation levels with the lowest SEs were selected for further evaluation tasks.

#### 3.1.2 Unsupervised segmentation methods

An improved version of the ESP tool (Drǎguţ et al., 2010) was available for this comparative evaluation. The new tool automatically identifies patterns in data at three different scales, ranging from fine objects (Level 1) to broader regions (Level 3), using a data-driven approach. The method relies on the ability of local variance (LV) to detect scale transitions in geospatial data (Drăguţ et al., 2014). Segmentation is performed with the MRS algorithm in a bottom-up approach in which the SP increases at a constant rate. The average LV of the objects in all layers is computed and serves as a condition for stopping the iteration: when a segmentation level records a LV value equal to or lower than the previous one, the iteration ends and the objects segmented in the previous segmentation level are retained. The method has been implemented as a customized process in eCognition® software and operates fully automatically, i.e., without any user intervention (Drăguţ et al., 2014). For the sake of clarity, we will refer to this as the ESP2 tool.

The SOP is an iterative method based on segmentation and classification refinement procedures (Esch et al., 2008). The method uses MRS to generate an initial segmentation level using a small SP pre-defined in the algorithm. Spectral statistics such as brightness are calculated for the generated image objects (designated sub-objects). A new segmentation level is then generated using a larger SP. The spectral similarity between the newly generated image objects (designated super-objects) and the previously generated sub-objects is quantified using the mean percentage difference in brightness (mPD_B_) and the mean percentage difference in spectral signature (mPD_R_). Those sub-objects with spectral statistics that exceed user-defined thresholds for either the mPD_B_ or the mPD_R_ are classified as distinct substructures (SubSts) and clipped from the super-objects. In addition, any adjacent SubSts with similar brightness values are merged at the super-objects level. Optional parameters such as the mean absolute difference in brightness (mB_BN_) between neighboring objects can be used to impose additional conditions on the sub-object classification. The segmentation optimization procedure runs until the largest objects to be identified in the image (e.g., agricultural fields) have been delineated (Esch et al., 2008).

The SOP method was implemented as an operational tool using the Definiens Architect®. We used the version of this tool that works on four image layers. For this study, we defined the same parameter thresholds as Esch et al. (2008), such as 0.7 for the mPD_B_. Optional parameters were ignored. The tool was applied to all of the QuickBird image layers. For Test Area B and Test Area C, we selected four of the eight available spectral bands in the WorldView-2 imagery, namely blue (band 2), green (band 3), red (band 5), and nir1 (band 6), these being the closest equivalents to the QuickBird bands.

### 3.2 Evaluating the segmentation results

The segmentation results were evaluated by comparing the geometries of the resulting image objects using the metrics introduced in Section 3.2.1 and by means of an accuracy assessment of the resulting building classifications (Section 3.2.2).

#### 3.2.1 Comparing the geometries of the image objects

The geometries of the image objects were compared by means of empirical discrepancy methods, also known as supervised segmentation evaluation (Zhang, 1996). These methods assess the geometric differences between the generated image objects and reference data. The image objects generated with the SAA method and classified as buildings were used as reference data for evaluating the geometry of the building objects identified using the ESP2 and SOP methods. In this way, we evaluated the ability of the two unsupervised methods to produce image objects that approach the geometries of the image objects created using the supervised method (Table 2). The area fit index (AFI), quality rate (Qr) and Root Mean Square (D_ij)_ are global metrics that take into account the entire imagery for evaluation purposes. The D_ij_ metric combines the undersegmentation (USeg) and oversegmentation (OSeg) metrics to evaluate the ‘closeness’ of the image objects to the reference data (Clinton et al., 2010). Using the Oseg and USeg metrics is referred to as local validation because “single objects are considered” (Möller et al., 2007). OSeg occurs when the image objects are smaller than the reference objects, and USeg occurs when the image objects are larger than the reference objects. In addition to using the metrics described above, we calculated the area of the overlapping segments, the number of misses (i.e., the number of objects identified as buildings in the reference data but missing from the evaluated segmentation layer), and the missing rate (i.e., the number of missing objects divided by the total number of objects in the reference data). In the ideal case of a perfect match between two segmentations, the AFI, OSeg, USeg and missing rate values would be zero, and the Qr would be 1. The metrics displayed in Table 2 were implemented in eCognition® software following Eisank et al. (2014).

**Table 2:** Metrics used for the evaluation of segmentation.

Metrics	Formula	Explanations	Authors
Over-segmentation (OSeg)	$\mathrm{OSeg}=1-\frac{\mathrm{area}(x_{i}\cap y_{j})}{\mathrm{area}(x_{i})}$*x_i_* – reference objects *y_j_* – evaluated objects	Range [0,1] OSeg = 0 → perfect segmentation	Clinton et al. (2010)
Under-segmentation (USeg)	$\mathrm{USeg}=1-\frac{\mathrm{area}(x_{i}\cap y_{j})}{\mathrm{area}(y_{i})}$	Range [0,1] USeg = 0 → perfect segmentation	Clinton et al. (2010)
Root mean square (D_ij_)	$D_{\mathrm{ij}}=\sqrt{\frac{{\mathrm{OSeg}}_{\mathrm{ij}}^{2}+{\mathrm{USeg}}_{\mathrm{ij}}^{2}}{2}}$	Range [0,1]; 0-perfect match	Levine and Nazif, 1985, Weidner, 2008
Area fit index (AFI)	$\mathrm{AFI}=\frac{\mathrm{area}(x_{i})-\mathrm{area}(y_{i})}{\mathrm{area}(x_{i})}$	AFI = 0.0 → perfect overlap	Lucieer and Stein (2002)
Quality rate (Qr)	$\mathrm{Qr}=\frac{\text{area}(X_{i}\cap y_{i})}{\text{area}(X_{i}\cup y_{i})}$	Range [0,1]; Qr 1 → perfect match	Winter (2000)

#### 3.2.2 Classification

For the classification, we used the random forest (RF) classifier (Breiman, 2001). This classifier requires the definition of two parameters (Immitzer et al., 2012): (1) the number of classification trees and (2) the number of input variables considered at each node split. On the basis of previous research (Breiman, 2001, Duro et al., 2012a, Immitzer et al., 2012), we selected 500 trees and √m variables at each split (where *m* represents the number of variables). The RF classifier has been extensively used for different classification tasks because of its predictive power and because it allows the importance of the features used to classify the target objects, known as the variable importance (VI), to be calculated (Corcoran et al., 2013, Guo et al., 2011, Stumpf and Kerle, 2011). The RF classifier was applied using the R statistical analysis package (R-Development-Core-Team, 2005).

Independent sets of 57 image features (attributes) were computed for the image objects in each of the evaluated test areas and used as variables in the RF classifier. These attributes included spectral information (mean values, ratios and standard deviations), indexes (the normalized difference vegetation index, and the normalized difference water index), geometric information (shape and extent metrics) and 25 textural parameters (Haralick, 1979).

##### 3.2.2.1 Training and validation samples

To generate the training and validation samples, we developed catalogues of buildings for each of the test areas using OSM buildings data (see Section 3.1.1 for information on OSM data). In this catalogue, the buildings were pre-classified into six classes according to the spectral reflectance and associated color of their roofs: (1) bright-gray roofs, (2) dark-gray roofs, (3) bright-red roofs, (4) dark-red roofs, (5) green roofs or (6) blue roofs. Stratified training samples were then generated for each “building class” (BC), aiming at equal representation for each of the six sub-classes. The remaining land cover classes were grouped under the heading of “other class” (OC). The reference data for the OC were randomly generated across the test areas, after first having masked out the buildings. Because each OSM reference polygon might intersect more than one image object, the centroids of the OSM reference polygons were used to select the samples from the image objects delineated by the ESP2, SAA and SOP methods in the three test areas.

The classifications results were assessed using a standard confusion matrix (Congalton, 1991). Three validation data sets were collected for the three test areas (Table 3). We generated 85 samples per building class in a stratified random sampling scheme using OSM buildings data. The 85 samples for the OC were randomly generated after first masking out the buildings within the study areas.

**Table 3:** Summary of the reference data: number of training samples used to train the RF classifier and of validation data used to validate the classification accuracy of the “buildings class” (BC) and “other classes” (OC).

Test area	Imagery	Training data		Validation data	
		BC	OC	BC	OC
A	QuickBird	128	164	85	85
B	WorldView-2	107	104	85	85
C	WorldView-2	130	134	85	85

The differences between classifications were assessed by comparing the Kappa indexes (Congalton, 1991) and the overall accuracies. The data used to train the RF classifier differed from the data used to assess the accuracy of the resulting building classifications.

## 4 Results

The optimal SPs, as estimated by the ESP2 and SAA methods, are shown in Table 4. There is no SP for the SOP because this method generates a single image segmentation layer by fusing together the image objects obtained with different SPs (Esch et al., 2008). The SAA and ESP2 methods unexpectedly estimated surprisingly similar SPs (as seen in Table 4), the difference between them ranging from 2 to 23 for small buildings and from 0 to 59 for large buildings. Because there is no SP for the SOP, its outputs were evaluated using the differences in the number of objects. The number of image objects generated by the SOP was much lower than the number of image objects generated by the other two methods (Table 5). This result is not surprising given that the SOP is a segmentation optimization procedure that generates image objects through a sequence of clipping and merging techniques.

**Table 4:** Overview of optimal SPs estimated using the SAA and ESP2 tools.

Scale parameter	Test area A		Test area B		Test area C	
	Small buildings	Large buildings	Small buildings	Large buildings	Small buildings	Large buildings
SAA	110	491	150	341	170	490
ESP2	133	491	152	400	186	501

**Table 5:** Number of image objects obtained for the three test areas using each of the three approaches.

	Image Objects		
	Test area A	Test area B	Test area C
SAA	9083	5501	7060
ESP2	6549	5398	6030
SOP	3918	3848	6491

The image objects used for the further evaluations were generated using the SP for small buildings estimated by the SAA method, the finest segmentation level produced by the ESP2 method, and the level generated by the SOP.

Table 6 reveals a marked discrepancy between the results obtained using the SAA method and those obtained using the SOP, as well as a large overlap between the image objects generated using the SAA method and those generated using the ESP2 method. The overlap threshold was set to 0.5, which was considered appropriate for matching objects when assessing segmentation goodness (Zhan et al., 2005). The SAA results were used as reference data for the evaluation of the two unsupervised methods.

**Table 6:** Segmentation evaluation metrics. The objects generated as buildings using the SAA tool were used as reference data for evaluating the building objects generated by the ESP2 and SOP tools. Detailed explanations of the segmentation evaluation metrics are provided in Table 2.

	No. ref	AFI	D_ij_	Missing rate	No. of misses	OSeg	Overlap (sq.m.)	Qr	USeg
Area A SAA vs. ESP2	4115	0.13	0.10	0.016	689	0.14	58,580,625	0.84	0.01
Area A SAA vs. SOP	4115	0.64	0.46	0.63	2600	0.66	23,324,875	0.33	0.05
Area B SAA vs. ESP2	3142	0.02	0.01	0.01	40	0.02	83,696,550	0.97	0.0004
Area B SAA vs. SOP	3142	0.52	0.39	0.53	1693	0.54	38,570,675	0.43	0.062
Area C SAA vs. ESP2	3976	0.09	0.07	0.06	239	0.10	137,576,800	0.88	0.007
Area C SAA vs. SOP	3976	0.53	0.39	0.42	1681	0.55	69,163,825	0.44	0.039

The segmentation evaluation metrics show a near perfect match between the geometries of the image objects obtained using the SAA method and those obtained using the ESP2 method. Comparing these two methods revealed optimal AFIs and Qr values for Test Area B (0.02 and 0.97, respectively) and Test Area C (0.09 and 0.88, respectively). The USeg and OSeg values were also optimal (Table 6). The AFI for Test Area A was 0.13, and the Qr value was 0.84. These results show that the SAA and ESP2 methods performed equally well for different areas, as well as on data acquired with different sensors (i.e., the WorldView-2 and QuickBird sensors).

In contrast, the segmentation evaluation metrics indicated a larger discrepancy between the SAA and SOP methods in delineating buildings. Thus, the AFI revealed a lower degree of fitness between SAA and SOP image objects (0.64 for Test Area A, 0.52 for Test Area B, and 0.53 for Test Area C). The OSeg and USeg values also increased, generating D_ij_ values of 0.46 for Test Area A and 0.39 for Test Area B and C. Thus, the Q_r_ yielded modest values between 0.33 and 0.44.

**Fig. 2:**
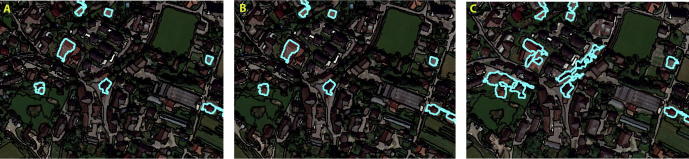
Segmentation results for a subset of test area B. (A) SAA; (B) ESP2; (C) SOP (true color composition). (For interpretation of the references to colour in this figure legend, the reader is referred to the web version of this article.)

**Fig. 3:**
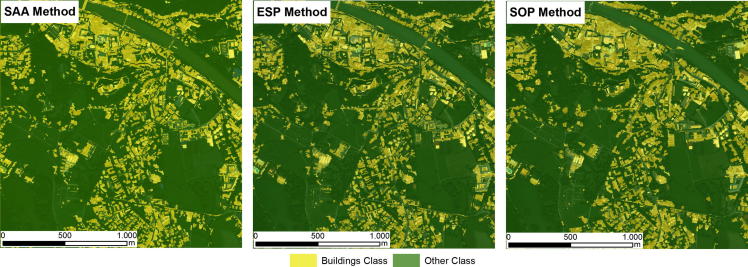
Building classification results for test area A.

**Fig. 4:**
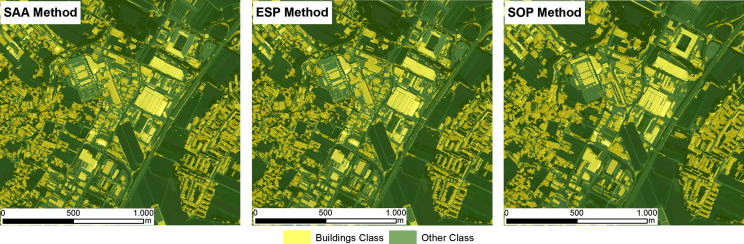
Building classification results for Test Area B.

**Fig. 5:**
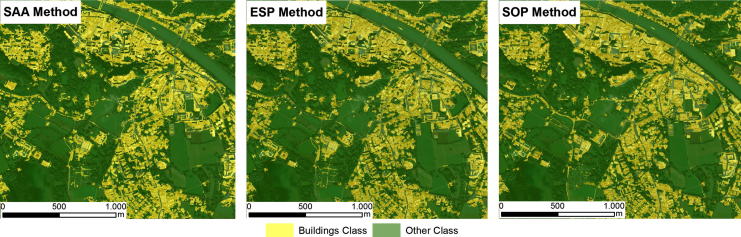
Building classification results for test area B.

Because the evaluated methods all generated different image objects, the RF classifier generated slightly different classification models for each of the evaluated methods. Thus, the VI of the features used to classify the target objects varied across the different evaluated methods. The results of the classifications in the test areas are shown in Fig. 3, Fig. 4, Fig. 5. The accuracies of the building classifications based on the image objects generated by the three evaluated methods were surprisingly similar, with overall accuracies (OAs) ranging from 82.3% to 86.4%, and Kappa coefficients ranging from 0.64 to 0.72 (Table 7). In Test Area A, the SOP slightly outperformed the SAA and ESP2 methods, achieving an overall accuracy of 84.1% (Kappa coefficient: 0.68). The SAA and ESP2 methods yielded the same overall accuracy of 83.5% (Kappa coefficient: 0.67). In Test Area B, the ESP2 method achieved an overall accuracy of 85.2% (Kappa coefficient: 0.7), the SAA method yielded an overall accuracy of 84% (Kappa coefficient: 0.68), and the SOP yielded an overall accuracy of 82.3% (Kappa coefficient: 0.64). The latter achieved a slightly lower accuracy than the SAA and ESP2 methods, mainly because the SOP tends to under-segment small buildings, as shown in Fig. 2. In Test Area C, the SOP and ESP2 methods achieved the same overall accuracy (86.4%) and Kappa coefficient (0.72), whereas the SAA method achieved an overall accuracy of 84.1% (Kappa coefficient: 0.68), almost identical to the results achieved by the same method in the other test areas. All three classifications models appeared to be insensitive to the change of sensor, as shown by overall accuracies that are almost identical for test areas A and C (Table 7, and Fig. 3, Fig. 4, Fig. 5).

**Table 7:** Overall Accuracies (OA) and Kappa coefficients (Kappa) yielded by the SAA, ESP2 and SOP methods for the three evaluated test areas.

	Test area A			Test area B			Test area C		
	SAA	ESP 2	SOP	SAA	ESP2	SOP	SAA	ESP2	SOP
OA (%)	83.5	83.5	84.1	84.1	85.2	82.3	84.1	86.4	86.4
Kappa	0.67	0.67	0.68	0.68	0.7	0.64	0.68	0.72	0.72

## 5 Discussion

The objective of this study was to compare supervised and unsupervised approaches in multiresolution segmentation. The performances of the three segmentation methods used in this study were evaluated by assessing the classification accuracy and by comparing the geometries of the resulting image objects.

The experiments showed that the results from the two unsupervised methods were remarkably similar to those from the supervised method (Fig. 3, Fig. 4, Fig. 5), especially in terms of their thematic accuracy (Table 7). These results are counter-intuitive, as one would expect superior results from the supervised method. Because supervised segmentation is guided by additional information about target classes via the geometry of samples, it is reasonable to expect that it would be best able to tune the image objects to the desired outputs. Therefore, we assumed that supervised segmentation would always produce more accurate results than unsupervised methods and attempted to evaluate the magnitude of the differences. However, our results have shown that unsupervised segmentation can be successfully used to extract buildings from the satellite imagery employed in our tests, instead of a more tedious supervised method, and that the resulting gain in automation is not accompanied by any loss in thematic accuracy.

The results of the evaluation of the geometries were even more surprising, as they showed a close match between the image objects generated using the SAA method and those generated using the ESP2 method (Table 6). In view of the strong differences between supervised and unsupervised methods, one would expect differences in the geometries of generated image objects, as is the case with those generated using the SAA and SOP methods (Table 6). Although differences were expected between the SAA and SOP methods, it is surprising that the different objects led to very similar classifications (Table 7 and Fig. 3, Fig. 4, Fig. 5), which appears to challenge the belief that has been expressed more or less explicitly since the earliest use of image segmentation applications in remote sensing (e.g., Ton et al., 1991, Woodcock and Harward, 1992) that the results of segmentation have a marked impact on classification accuracy. The effect of image segmentation on the classification accuracy was recently investigated by Gao et al. (2011), who confirmed that the best accuracy was obtained using optimal segmentation and that both over-segmentation and under-segmentation led to less accurate results. However, our own results suggest that classification accuracy is significantly less dependent on segmentation results, at least when extracting buildings from VHR imagery.

While the SOP produced very different objects from the SAA method, most of the ‘building’ objects were over-segmented, and a few of them were under-segmented (Table 6). The numerous image objects corresponding to a building may have been merged in the classification step. Although over-segmentation is preferable to under-segmentation (Castilla and Hay, 2008, Gao et al., 2011, Marpu et al., 2010, Neubert et al., 2006), it still leads to a lower accuracy than ‘optimal segmentation’ (Gao et al., 2011). However, if we consider SAA segmentation to be optimal, the SOP still resulted in superior accuracy in two of the three cases (Table 7). On the basis of these results, we suggest that there is no such thing as ‘optimal segmentation’ and that as long as under-segmentation remains at acceptable levels, imperfections in segmentation can be ruled out, and a high level of classification accuracy can still be achieved.

Schiewe (2002) noted that “most of the semi-automatic object recognition procedures do not lead to satisfactory results” (p. 386). However, our results show that the three different methods that we tested performed very well when used for extracting buildings from VHR imagery, although the relative importance of segmentation and classification in achieving the reported high accuracies remains unclear. For the classifications, we used the RF classifier, which is a non-parametric ensemble learning classifier (Breiman, 2001) that has been successfully used for mapping landslides (Stumpf and Kerle, 2011) or land cover classes (Corcoran et al., 2013, Duro et al., 2012b, Gislason et al., 2006, Guan et al., 2013, Rodriguez-Galiano et al., 2012). The use of the RF classifier in this study appears to have made an important contribution to the thematic accuracy. The classifier was able to compensate for the differences between the geometries of objects generated using the SAA method and those generated using the SOP by assigning different weightings to the features used in the classification: where image objects were over-segmented, shape features were replaced by spectral information. The dependence of the VI on the segmentation scale has previously been demonstrated by Stumpf and Kerle (2011).

Because the three methods performed similarly in this evaluation exercise, it may be possible to discriminate between them on the basis of their usability and potential for automation. The SOP requires the input of several user-defined parameters, which control the number, size and geometry of the image objects. It works on a maximum of five image layers; therefore, a further extension is required to accommodate the increasing spectral resolution of WorldView-2 and other forthcoming satellite products.

The SAA method relies on reference data, and the collection of such data increases the overall time required for image classification. This method was originally used to map geomorphological features (Anders et al., 2011). In that particular study, three reference data were generated for each geomorphological unit, but in our case, a larger number of reference data were required, given the high level of within-class variation in the building objects and the larger areal extent covered by the analyzed imagery. However, further tests will be required to assess the sensitivity of the SAA method to variations in the number of samples.

In contrast to the SAA and SOP methods, the ESP2 does not require any human intervention to set segmentation parameters. The ESP2 tool identifies patterns in the underlying data on multiple levels using only the statistics from the image objects and works on up to 30 image layers with the number of input layers being detected automatically (Drăguţ et al., 2014).

The supervised segmentation methods are good at identifying the correct SP for the target objects. However, their dependence on reference data makes them less easy to use in operational settings than the unsupervised methods (Zhang et al., 2008). The unsupervised methods are, in contrast, less subjective and more time-efficient, making them suitable for use in operational satellite imagery classification settings.

As has been previously stated by Hay and Castilla, 2008, Arvor et al., 2013, the semantic gap between the image objects and the real-world geographic objects (geo-objects) challenges the task of image classification. A model-based classification that formalizes the properties of real-world objects and their representation in imagery cannot perform well because optimal image objects (approximate the classes of interest) are very difficult to obtain (Lang, 2008). A supervised segmentation would be the most intuitive approach with which to address this problem, but this study has shown that supervised approaches do not outperform unsupervised approaches, at least for building classification. A possible alternative would be to combine unsupervised segmentation with supervised classification (using, for instance, RF classifier or another similar classifier) of the image objects, followed by similarity measurements between the resulting classified image objects and geo-objects whose characteristics are explicitly formalized in object libraries (Strasser et al., 2013) or ontologies (Arvor et al., 2013, Kohli et al., 2012).

## 6 Conclusions

This study sought to investigate and compare supervised and unsupervised segmentation approaches in OBIA by using them to classify buildings from three test areas in Salzburg, Austria, using QuickBird and WorldView-2 imagery. In our investigations, we used the SAA supervised segmentation method and two unsupervised methods (SOP and ESP2). All three of the methods evaluated achieved remarkably similar classification accuracies for our test areas, with overall accuracies between 82.3% and 86.4% and Kappa coefficients between 0.64 and 0.72. Because supervised segmentation requires a prohibitive amount of effort (and time), unsupervised methods may offer an important alternative that will improve the applicability of OBIA in operational settings due to their greater degree of automation.

Our investigations have also revealed unexpected similarities in the segmentation results from the supervised method and those from one of the unsupervised methods (the ESP2 tool). The two methods identified almost identical SPs as optimal for segmenting buildings, which led to very similar geometries for the resulting image objects.

The results from our comparison of the SAA and SOP methods challenge previous findings that segmentation has a marked impact on classification: although the two approaches produced very different image objects, their classification accuracies were very similar. This result suggests that, as long as under-segmentation remains at acceptable levels, imperfections in segmentation can be ignored so that a high level of classification accuracy can still be achieved.
